# Intersubband plasmons in the quantum limit in gated and aligned carbon nanotubes

**DOI:** 10.1038/s41467-018-03381-y

**Published:** 2018-03-16

**Authors:** Kazuhiro Yanagi, Ryotaro Okada, Yota Ichinose, Yohei Yomogida, Fumiya Katsutani, Weilu Gao, Junichiro Kono

**Affiliations:** 1 Department of Physics, Tokyo Metropolitan University, Tokyo, 192-0397 Japan; 2 Department of Electrical and Computer Engineering, Rice University, Houston, TX 77005 USA; 3 Department of Physics and Astronomy, Rice University, Houston, TX 77005 USA; 4 Department of Materials Science and Nanoengineering, Rice University, Houston, TX 77005 USA

## Abstract

Confined electrons collectively oscillate in response to light, resulting in a plasmon resonance whose frequency is determined by the electron density and the size and shape of the confinement structure. Plasmons in metallic particles typically occur in the classical regime where the characteristic quantum level spacing is negligibly small compared to the plasma frequency. In doped semiconductor quantum wells, quantum plasmon excitations can be observed, where the quantization energy exceeds the plasma frequency. Such intersubband plasmons occur in the mid- and far-infrared ranges and exhibit a variety of dynamic many-body effects. Here, we report the observation of intersubband plasmons in carbon nanotubes, where both the quantization and plasma frequencies are larger than those of typical quantum wells by three orders of magnitude. As a result, we observed a pronounced absorption peak in the near-infrared. Specifically, we observed the near-infrared plasmon peak in gated films of aligned single-wall carbon nanotubes only for probe light polarized perpendicular to the nanotube axis and only when carriers are present either in the conduction or valence band. Both the intensity and frequency of the peak were found to increase with the carrier density, consistent with the plasmonic nature of the resonance. Our observation of gate-controlled quantum plasmons in aligned carbon nanotubes will not only pave the way for the development of carbon-based near-infrared optoelectronic devices but also allow us to study the collective dynamic response of interacting electrons in one dimension.

## Abstract (web summary)

Quantum confinement has enabled the development of modern optoelectronic devices, including the quantum cascade laser, based on the control of intersubband plasmons. Here, Yanagi et al. observe intersubband plasmons in gated and aligned carbon nanotubes with applications in carbon-based optoelectronics and fundamental physics.

## Introduction

Intersubband absorption and emission result from resonant optical transitions between quantized subbands within the conduction or valence band of a quantum-confined semiconductor^1^. They have been well established in semiconductor quantum wells (QWs)^1–9^, serving as the physical basis^10–14^ for QW infrared photodetectors^10^ and quantum cascade lasers^11^.

An intersubband transition can occur only when the sample is doped so that there are charge carriers of a single type (either electrons or holes). These charge carriers are free fermions, partially occupying a continuum of states in the lowest-energy subband from the subband bottom to the Fermi energy. Therefore, in *k*-space, the lowest-energy subband has a well-defined Fermi wave vector *k*_F_, and a superposition of all *k*-conserving (or vertical) transitions within the |*k*| ≤ *k*_F_ range between the lowest subband and a higher-lying empty subband is observed as an absorption peak. As such, it is evident that an intersubband transition consists of a collection of single-particle transitions occurring at different points in *k*-space. It has been shown that intersubband transitions in QWs are intrinsically collective in nature, involving many interacting and quantum-confined electrons, and cannot be described by a single-particle model^1–4^. As a result, they are generally referred to as intersubband plasmons (ISBPs)^5–9^, for which both plasmonic effects and quantum confinement effects are equally important in determining the resonance frequency. This situation is distinctly different from plasmons in gold nanoparticles^12^ or graphene^13, 14^, where the quantum confinement energy is negligibly small compared to the plasmon energy.

There is much interest in exploring ISBPs in one-dimensional (1D) systems to further increase the accessible spectral range for applications and to probe many-body dynamics of interacting 1D electrons. Unlike the case of QWs, ISBPs in 1D structures can be excited with normal incidence light, significantly simplifying the required experimental geometries. There have been reports on observations of far-infrared ISBPs in lithographically defined semiconductor quantum wires^15^, but there has been no convincing evidence for ISBPs in nanomaterials such as single-wall carbon nanotubes (SWCNTs). Previous studies of heavily doped SWCNTs have revealed a new absorption band^16–19^, but the nanotubes were randomly oriented, which precluded definitive interpretation. Here, we present unambiguous evidence for ISBPs in gated and aligned SWCNTs through polarization-dependent absorption spectroscopy, elucidating the origin of the observed unknown feature in these previous studies on doped SWCNTs.

## Results

### Allowed transitions in semiconductor QWs and SWCNTs

**Fig. 1:**
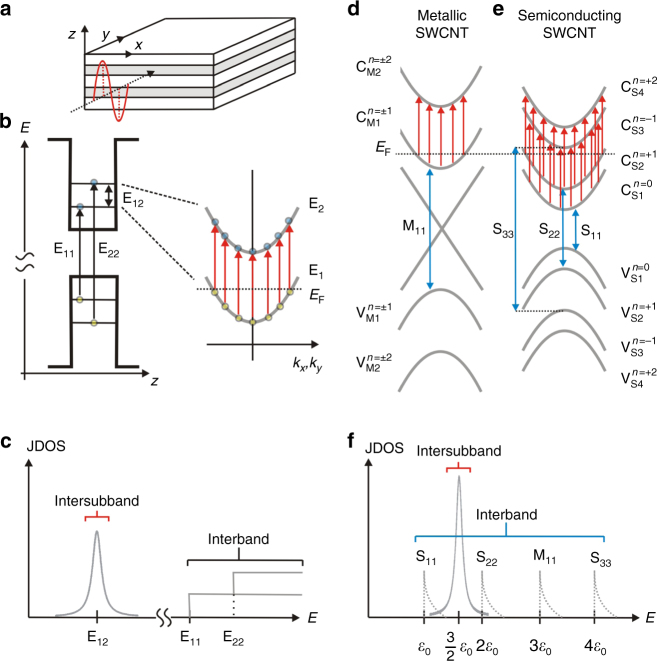
Schematic illustrations of allowed intersubband and interband transitions in semiconductor quantum wells and carbon nanotubes. **a** A quantum well made from a heterostructure of two semiconductors (white and gray) with different band gaps. **b** Intersubband (E_12_) and interband (E_11_ and E_22_) transitions in a quantum well. E_12_ is allowed only for incident light polarized along the quantum-confinement (*z*) direction and only when the system is doped (*n*-doped in the present case). **c **The joint density of states (JDOS) for the intersubband and interband transitions in a quantum well. Interband transitions (blue arrows) and intersubband transitions (red arrows) in **d** metallic and **e** semiconducting carbon nanotubes. See the text for the selection rules applied to these transitions. **f** The JDOS for the intersubband and interband transitions in carbon nanotubes. Here, *ε*_0_ = 2*γ*_0_*a*_C_ _−_ _C_/*d*_t_, *γ*_0_ is the nearest-neighbor transfer integral for graphene, *a*_C_ _−_ _C_ is the nearest neighbor C-C separation in graphene, and *d*_t_ is the nanotube diameter

In the ideal case of a semiconductor QW (depicted in Fig. 1a) where the two subbands are parallel in in-plane (*x*–*y*) dispersions (Fig. 1b), the joint density of states has a single symmetric peak (Fig. 1c), which leads to strong resonant absorption when the incident photon energy coincides with the subband separation and the radiation is polarized in the growth (*z*) direction. The resonance energy is independent of the band gap of the semiconductor material and thus fully quantum designable.

Figure 1d, e schematically shows the band structure of metallic and semiconducting SWCNTs, respectively, with representative allowed optical transitions indicated by arrows. Conduction and valence subbands in metallic (semiconducting) tubes are indicated as C_M*i*_ and V_M*i*_ (C_S*i*_ and V_S*i*_), respectively, where *i* = 1, 2, 3, …. Each subband has a well-defined angular momentum along the tube axis with quantum number *n*. Light with polarization parallel to the nanotube axis can cause transitions with *n* = 0, whereas light with perpendicular polarization can cause transitions with *n* = ±1 (refs. ^20, 21^); see Supplementary Note 1 for more details on selection rules. In undoped SWCNTs, optical absorption spectra are dominated by *i*-conserving transitions (for example, the M_11_, S_11_, and S_22_ transitions), which are excited by light with parallel polarization^21^. In doped SWCNTs with Fermi energy (*E*_F_) inside the conduction (valence) band, light with perpendicular polarization is expected to excite the C_M1_ → C_M2_ (V_M1_ → V_M2_) transition in metallic SWCNTs and the C_S1_ → C_S3_ and C_S2_ → C_S4_ (V_S1_ → V_S3_ and V_S2_ → V_S4_) transitions in semiconducting SWCNTs. Note that the perpendicular-polarized interband transitions (for example, V_S1_ → C_S2_ and V_S2_ → C_S1_) are suppressed by a strong depolarization effect^22, 23^, while intersubband transitions are expected to be still strong due to the concentrated joint density of states, similar to the QW case (a delta function in the limit of two parallel parabolic bands); see Fig. 1c, f.

### Response to parallel-polarized light

**Fig. 2:**
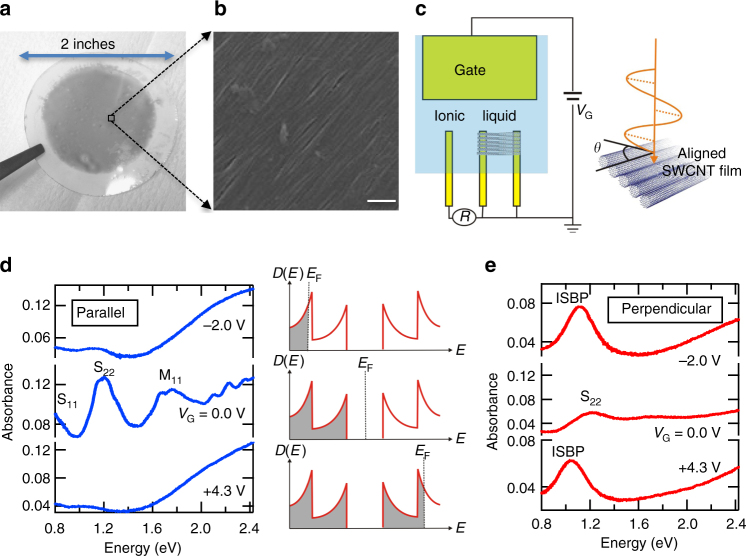
Appearance of the intersubband plasmon (ISBP) peak in a gated and aligned carbon nanotube film in polarized absorption spectra. **a** Optical and **b** scanning electron microscopy images of an aligned SWCNT film. The white horizontal bar in **a** represents a length of 100 nm. **c** Schematic diagram for the setup for the polarization-dependent absorption spectroscopy experiments on an aligned SWCNT film gated through electrolyte gating. *V*_G_: gate voltage, *R*: reference voltage. **d** Parallel-polarization and **e** perpendicular-polarization optical absorption spectra at different gate voltages for an aligned SWCNT film containing semiconducting and metallic nanotubes with an average diameter of 1.4 nm. The ISBP peak appears only for perpendicular polarization (**e**) for both electron and hole doping, corresponding to *V*_G_ = −2.0 and 4.3 V, respectively

We prepared films of macroscopically aligned and packed SWCNTs using vacuum filtration^24^. Optical and scanning electron microscopy images of a typical film are shown in Fig. 2a, b, respectively. We tuned *E*_F_ using electrolyte gating techniques^16, 17, 25, 26^. A schematic illustration of the experimental setup is shown in Fig. 2c; see Methods for more details. Figure 2d shows parallel-polarization optical absorption spectra for an aligned SWCNT film with an average diameter of 1.4 nm, containing both metallic and semiconducting SWCNTs, at three gate voltages, *V*_G_ = −2.0, 0.0, and +4.3 V. At *V*_G_ = 0.0 V, the V_S1_ → C_S1_ and V_S2_ → C_S2_ interband transitions in semiconducting nanotubes (labeled S_11_ and S_22_, respectively) and the V_M1_ → C_M1_ interband transition in metallic nanotubes (labeled M_11_) are clearly observed. As the gate voltage is increased in the positive (negative) direction, electrons (holes) are injected into the nanotubes through formation of an electric double layer on their surfaces. As a result, the S_11_, S_22_, and M_11_ peaks disappear at *V*_G_ = −2.0 and +4.3 V because of Pauli blocking.

### Response to perpendicular-polarized light

**Fig. 3:**
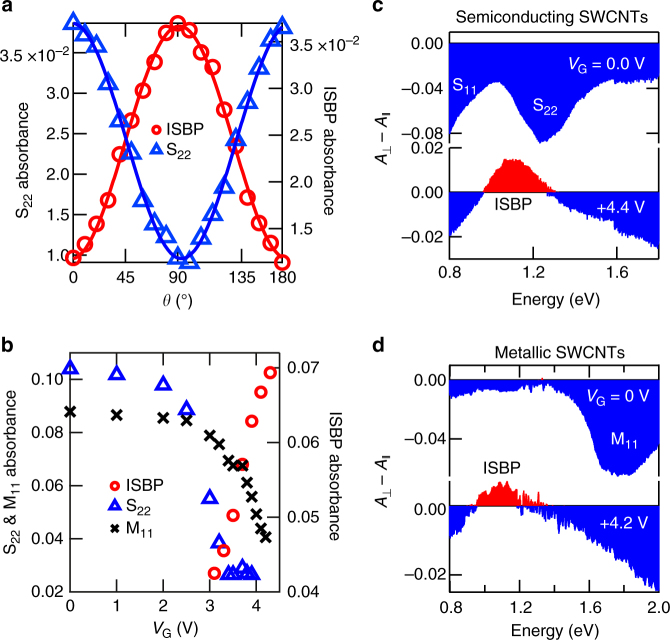
Opposite polarization and gate voltage dependence between the interband (S_22_ and M_11_) and intersubband (ISBP) absorption peaks. **a** Peak absorbance of the S_22_ peak (blue open triangles) and the ISBP peak (red open circles) as a function of polarization angle *θ*. The solid lines are sinusoidal fits to the data. **b** Peak absorbance of the S_22_ peak (blue open triangles), the M_11_ peak (black multiplication signs), and the ISBP peak (red open circles) as a function of gate voltage. Linear dichroism spectra for aligned **c** semiconducting and **d** metallic SWCNTs with an average diameter of 1.4 nm at various gate voltages. The S_11_, S_22_, and M_11_ peaks have negative (blue) signals whereas the ISBP appears as a positive (red) peak

Completely different behavior is observed in perpendicular polarization spectra, as shown in Fig. 2e. As |*V*_G_| is increased, a new absorption peak (labeled ISBP) appears at around 1.0 eV, grows in intensity, and dominate the spectrum at the highest |*V*_G_|; see Supplementary Figure 1 for more detailed gate-voltage-dependent spectra. The properties of the ISBP peak are entirely orthogonal to those of the S_11_, S_22_, and M_11_ peaks in two distinct aspects. First, as shown in Fig. 3a, the intensities of the latter become maximum for light with polarization parallel to the nanotube axis, whereas the intensity of the ISBP peak becomes maximum for light with perpendicular polarization. Second, upon carrier injection (either by electrons or holes), the S_22_ and M_11_ peaks shrink in intensity and eventually disappear, whereas the ISBP peak appears and quickly grows, as shown in Fig. 3b. This behavior highlights the fact that the density of carriers (either electrons or holes) has to be high enough to Pauli block the S_22_ and M_11_ peaks for the ISBP peak to be observable.

### Metal- and semiconductor-enriched SWCNT films

Furthermore, in order to determine whether the ISBP peak arises from semiconducting or metallic nanotubes (or both), we prepared films of aligned and type-separated SWCNTs (see Methods). Figure 3c, d shows linear dichroism spectra for aligned semiconducting and metallic SWCNTs, respectively, with an average diameter of 1.4 nm at zero and finite gate voltages. Here, the perpendicular-polarization absorbance, *A*_⊥_, minus the parallel-polarization absorbance, $A_\parallel$, namely $A_ \bot - A_\parallel$, exhibits negative signal for the S_11_, S_22_, and M_11_ peaks and positive signal for the ISBP peak, both in the semiconducting and metallic films. The peak position is essentially the same for both types of SWCNTs with the same average diameter. (Linear dichroism spectra for the semiconductor–metal mixture SWCNT film are shown in Supplementary Figure 2, and polarization-dependent absorption spectra for semiconducting and metallic SWCNTs are described in Supplementary Figures 3 and 4, respectively.)

### Gate-dependent changes of the ISBP peak

**Fig. 4:**
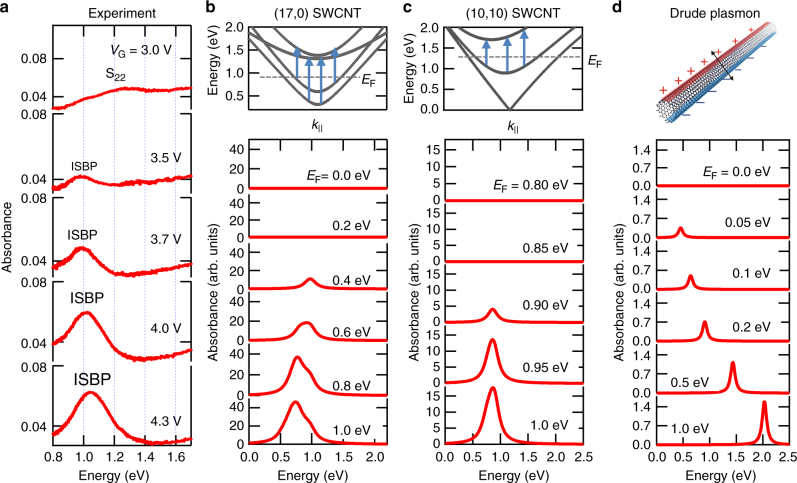
Experimental spectra and theoretical simulations for the electron density dependence of the intersubband plasmon peak. **a** Optical absorption spectra showing how the ISBP peak evolves as the gate voltage increases. **b** Calculated single-particle absorption spectra for a (17, 0) semiconducting nanotube at different Fermi energy values. **c** Calculated single-particle absorption spectra for a metallic (10, 10) nanotube at different Fermi energy values. **d** Calculated perpendicular-polarization absorption spectra for a SWCNT with a diameter of 1.4 nm at different Fermi energy values based on a classical Drude plasmon model

Figure 4a shows detailed changes of the ISBP peak for the aligned film made of a mixture of semiconducting and metallic SWCNTs as the gate voltage is varied on the electron injection side. The peak first appears at around 1 eV when the gate voltage reaches  approximately 3 V, and then its intensity gradually increases with increasing *V*_G_. In addition, the peak position blue shifts with further increasing *V*_G_, reaching 1.05 eV at 4.3 V. To provide more insight into the microscopic mechanism of this behavior, observed only for perpendicular polarization, we present our theoretical considerations and simulations below.

## Discussion

We start with simulations of absorption spectra based on single-particle transition energies. According to the simplest tight-binding model with the linear-*k* approximation for the electronic structure of SWCNTs^27^, conventional parallel-polarization interband transition energies can be written as *E*_IB,*j*_ = *ε*_0_*j*, where *ε*_0_ ≡ 2*γ*_0_*a*_C__ −_ _C_/*d*_t_, *γ*_0_ (~2.9 eV) is the nearest-neighbor transfer integral, *a*_C_ _− __C_ (=0.142 nm) is the nearest-neighbor C–C separation in graphene, *d*_t_ is the nanotube diameter, *j* = 1, 2, 4, and 5 for the S_11_, S_22_, S_33_, and S_44_ transitions, respectively, and *j* = 3 and 6 for the M_11_ and M_22_ transitions, respectively. Within this linear-*k* approximation, the transition energies for the C_S1_ → C_S3_, C_S2_ → C_S4_, V_S1_ → V_S3_, and V_S2_ → V_S4_ intersubband transitions allowed for semiconducting SWCNTs and for the C_M1_ → C_M2_ intersubband transition allowed for metallic SWCNTs are universally given by

1 $$E_{{\mathrm{ISB}}} = \frac{3}{2}\varepsilon _0.$$

Supplementary Note 1 provides a description of the energies and selection rules for intersubband transitions. Equation (1) indicates that the single-particle intersubband transition energy is essentially independent of metallicity and subband indices. The energy depends only on the nanotube diameter. This universal transition energy is consistent with our observation that the ISBP peak position for both the semiconducting and metallic films with the same diameter was at around 1 eV. Specifically, for *d*_t_ = 1.4 nm, Eq. (1) provides an estimate *E*_ISB _= 0.9 eV.

To compute single-particle absorption spectra within the tight-binding approximation^28^, we used the following formula^29^:

2 $$A(\omega ) = A{\int}_{\hskip-4pt 0}^\infty \rho (\omega _{{\mathrm{if}}}){\rm d}\omega _{{\mathrm{if}}}[f_{{\mathrm{FD}}}(\omega _{\mathrm{i}}) - f_{{\mathrm{FD}}}(\omega _{\mathrm{f}})]\frac{{1/\pi T_2}}{{(\omega - \omega _{{\mathrm{if}}})^2 + 1/T_2^2}}.$$

Here, *A* is a constant, *ρ* is the joint density of states of the two subbands under consideration (i and f) at a given transition frequency *ω*_if_ = *ω*_f_ − *ω*_i_, *f*_FD_ is the Fermi-Dirac distribution function for a given *E*_F_, and *T*_2_ is a phenomenological relaxation time to take account of line broadening. Figure 4b, c shows calculated absorption spectra for (17,0) and (10,10) SWCNTs, respectively, for five different values of *E*_F_ with $\hbar /T_2$ = 0.1 eV; see Supplementary Figures 5 and 6 for more details. In both cases, we can see that the absorption peak suddenly appears at a finite *E*_F_ and then rapidly grows, in agreement with experiment. However, the peak position does not show any blueshift. Note that the redshift seen for the (17,0) nanotube (Fig. 4b) is due to the slight energy difference between the C_S1_ → C_S3_ and C_S2_ → C_S4_ transition energies introduced through the tight-binding model we used^28^, which provides more realistic subband separation energies than Eq. (1).

The failure of the single-particle model to explain the *E*_F_ dependence of the ISBP peak energy is reminiscent of the well-known manifestations of many-body effects on intersubband transitions in conventional semiconductor QWs. The first many-body correction brought by doping is the exchange and correlation effect, which tends to lower the energy of the partially filled lower subband with respect to the empty upper subband. Thus, the transition energy *E*_ISB_ increases to $E_{{\mathrm{ISB}}}^ \ast$. In addition to this static many-body correction, there are two types of dynamic correction, which further modifies the transition energy to^1–3^

3 $$E_{{\mathrm{ISBP}}} = E_{{\mathrm{ISB}}}^ \ast \sqrt {1 + \alpha - \beta } .$$

Here, *α* represents the depolarization shift due to the collective plasma oscillations of carriers induced by the electric field of light, and *β* represents the final state interaction (also known as the excitonic correction)^1–3^. The depolarization shift is a blueshift, while the excitonic effect induces a redshift. The appearance of the ISBP peak around 1.0 eV, which is higher than the single-particle transition energy (~0.9 eV), suggests that the static blueshift plus the dynamic blueshift (*α*) exceeds the redshift due to the excitonic effect (*β*).

Given that detailed many-body calculations of ISBP energies for doped SWCNTs are not available, we only provide estimates for the perpendicular polarization optical absorption within a classical Drude plasmon model for SWCNTs^30^, according to which the resonance energy is given by $\hbar \omega _{\mathrm{p}} = 2(\hbar cE_{\mathrm{F}}/137d_{\mathrm{t}})^{1/2}$; see more details of the calculations in Supplementary Note 4. Within this model, we calculated perpendicular-polarization absorption spectra for a SWCNT with *d*_t_ = 1.4 nm with various values of *E*_F_ and a broadening factor of 0.1 eV, as shown in Fig. 4d. It can be seen that this Drude plasmon model can reproduce a carrier-induced blueshift of the peak position, as expected. However, these calculated spectra fail to agree with our observed spectra in two critical aspects. First, the plasmon peak is observed even at small values of *E*_F_. Namely, the sharp turn-on of the absorption peak observed in our experiments is not reproduced. Second, the peak position moves with *E*_F_ too rapidly, reaching 2 eV at *E*_F_ = 1 eV.

Our observations of a charge-induced giant optical absorption peak in aligned SWCNTs for perpendicular polarized light thus create new challenges for theoretical studies. A correct theoretical model must describe the resonant interaction of light and electrons in the presence of extreme quantum confinement in the molecular limit^31^ and strong electron–electron interactions characteristic of 1D systems (described by the Tomonaga–Luttinger model)^32^^–40^. At the same time, the observed near-infrared (~1 eV) ISBPs expand the spectral range of possible optoelectronic devices based on intraband transitions in quantum-confined semiconductors for the generation and detection of electromagnetic radiation for spectroscopy, imaging, and communications.

## Methods

### Sample preparation

Aligned SWCNTs were prepared through the pressure-controlled vacuum filtration method^24^. SWCNTs were mono-dispersed in a deoxy cholate (DOC) 0.5% solution, and the solution was diluted to be DOC 0.025%. The diluted solution was used for the preparation of aligned SWCNT films. The films were formed on a polycarbonate filter membrane using a standard filtration system, but the filtration speed was controlled to be approximately 1 min/mL. The membrane filter was dissolved in a chloroform and acetone solution, and then the SWCNT film was transferred to a glass substrate on which gold electrodes were formed. SWCNTs with mixed types, produced by the arc-discharge method (P2-SWCNT, Carbon Solutions, Inc.) was used for the data shown in Fig. 2. Semiconducting SWCNTs (Fig. 3c) and metallic SWCNTs (Fig. 3d) were prepared through density-gradient purification techniques previously reported for SWCNTs produced by the arc-discharge method (ArcSO, Meijo Nanocarbon Co.)^41^

### Gate-dependent polarized optical absorption spectroscopy

Measurements were performed using a home-built optical measurement setup combined with a source-meter and digital-multimeter for carrier injections. An ionic liquid (*N*,*N*,*N-*trimethyl-*N*-propylammonium bis(trifluoromethane sulfonyl)-imide (TMPA-TFSI), Kanto Kagaku Co.) was used for electrolyte gating to inject carriers. All measurements were performed in vacuum at room temperature.

### Data availability

All relevant data are available from the authors.

## Electronic supplementary material

Supplementary Information(PDF 2749 kb)
