# Supplementary material for: Intersubband plasmons in the quantum limit in gated and aligned carbon nanotubes
Source: Nat Commun. 2018 Mar 16;9:1121. doi: 10.1038/s41467-018-03381-y (PMC5856781; doi:10.1038/s41467-018-03381-y)
Supplement: Supplementary file 1 — Supplementary Information(PDF 2749 kb) [file 41467_2018_3381_MOESM1_ESM.pdf]

Supplementary Information for

Intersubband Plasmons in the Quantum Limit in Gated and Aligned Carbon Nanotubes

Kazuhiro Yanagi,* Ryotaro Okada, Yota Ichinose, Yohei Yomogida, Fumiya Katsutani, Weilu Gao, and Junichiro Kono†

*yanagi-kazuhiro@tmu.ac.jp, †kono@rice.edu

Supplementary Note 1 Selection Rules for Perpendicular Polarized Transitions

Here we discuss the selection rules for optical transitions in carbon nanotubes for light polarized perpendicular to the nanotube axis, following Supplementary Ref. [1].

Characteristics of wavefunctions in carbon nanotubes

Within an effective mass description of electronic states in single-wall carbon nanotubes (SWCNTs), the envelope wavefunction of each state can be expressed as

$$F_{s,n,k}(\mathbf{r}) = \frac{1}{\sqrt{AL}}|s,n,k)\exp\left(i\kappa_n x + iky\right) \tag{1}$$

where

$$|s,n,k) = \frac{1}{\sqrt{2}}\begin{pmatrix} \frac{s(\kappa_n - ik)}{\sqrt{\kappa_n^2+k^2}} \\ 1 \end{pmatrix}, \tag{2}$$

$\mathbf{r} = (x, y)$, x is the coordinate along the circumference, y is the coordinate along the nanotube axis,

$$\kappa_n = \frac{2\pi}{L}\left(n - \frac{\nu}{3}\right) \tag{3}$$

is the (quantized) wavenumber along the circumference ($n = 0, \pm 1, \pm 2, ...$), k is the wavenumber along the nanotube axis, L is the length of the circumference, A is the length of the nanotube, $s = +1$ (-1) for the conduction (valence) band, and $\nu = N - M \pmod 3$ for a SWCNT with chirality (N,M).

Note that $\nu = 0$ (± 1) for a metallic (semiconducting) nanotube. Specifically, in the case of a (17,0) nanotube, for which $\nu = -1$,

$$\kappa_n = \frac{2\pi}{L}\left(n + \frac{1}{3}\right), \tag{4}$$

and in the case of a (10,10) nanotube, for which $\nu = 0$,

$$\kappa_n = \frac{2\pi}{L} n. \tag{5}$$

Furthermore, the energy of the state $F_{s,n,k}$ is given by

$$\varepsilon_{s,n}(k) = \frac{3sL}{4\pi}\varepsilon_0\sqrt{\kappa_n^2 + k^2}, \tag{6}$$

where, as defined in the main text,

$$\varepsilon_0 = \frac{2\gamma_0 a_{\text{C-C}}}{d_{\text{t}}} = \frac{2\pi\gamma_0 a_{\text{C-C}}}{L}, \tag{7}$$

γ_0 ($\sim$2.9 eV) is the the nearest-neighbor transfer integral for graphene, $a_{\text{C-C}}$ (= 0.142 nm) is the nearest neighbor C-C separation in graphene, and d_{t} is the nanotube diameter. For example, the band-edge ($k = 0$) energy of the lowest-lying conduction subband ($\text{C}_{\text{S1}}^{n=0}$) in a $\nu = -1$ (semiconducting) carbon nanotube is calculated to be

$$\varepsilon_{1,0}(0) = \frac{3L}{4\pi}\cdot\varepsilon_0\cdot\frac{2\pi}{L}\cdot\frac{1}{3} = \frac{\varepsilon_0}{2}. \tag{8}$$

At $k = 0$, the wavefunction is written as

$$F_{s,n,0} = \frac{1}{\sqrt{AL}}|s, n, 0)\exp\left(i\kappa_n x\right) \tag{9}$$

$$= \frac{1}{\sqrt{AL}}\frac{1}{\sqrt{2}}\begin{pmatrix} s\frac{\kappa_n}{|\kappa_n|} \\ 1 \end{pmatrix}\exp\left(i\kappa_n x\right). \tag{10}$$

Note that the value of $s\kappa_n/|\kappa_n|$ is either $+1$ or -1, which means that

$$|s, n, 0) = \frac{1}{\sqrt{2}}\begin{pmatrix} 1 \\ 1 \end{pmatrix} \text{ or } \frac{1}{\sqrt{2}}\begin{pmatrix} -1 \\ 1 \end{pmatrix}. \tag{11}$$

Furthermore, one can easily see that these functions are the eigenfunctions of the Pauli matrix

$$\hat{\sigma}_x = \begin{pmatrix} 0 & 1 \\ 1 & 0 \end{pmatrix} \tag{12}$$

with eigenvalue $\sigma_x = +1$ and -1, respectively. When the polarization of the incident light is perpendicular to the nanotube axis, the induced current density operator $\hat{J}_x$ is proportional to $\hat{\sigma}_x$, and thus, the conservation of σ_x leads to one of the determining factors for the selection rules, as shown below.

Selection rules

We confine ourselves to the case where the light field is linearly polarized in a direction perpendicular to the nanotube axis. In this case, the current density induced in the circumferential direction, expressed in operator form, is given by [**?**]

$$\hat{J}_x^l = -\frac{3eL}{4\pi\hbar}\varepsilon_0 \hat{\sigma}_x \exp\left(-i\frac{2\pi l}{L}x\right), \tag{13}$$

where $l = \pm 1$ correspond to the two circular polarization components of the electric field of light. The optical conductivity, calculated using the Kubo formula [**?**], is non-zero (i.e., there is finite absorption) only when the following transition matrix element is non-zero between the states $F_{s,n,k}$ and $F_{s',n',k}$:

$$D_{snk,s'n'k} = \int F^*_{s,n,k} \hat{J}_x^l F_{s',n',k} dx \tag{14}$$

$$= -\frac{3e\varepsilon_0}{4\pi\hbar A}(s,n,k|\hat{\sigma}_x|s',n',k) \int e^{i\left\{(\kappa_{n'}-\kappa_n)-\frac{2\pi l}{L}\right\}x} dx. \tag{15}$$

Note that we are considering only k-conserving transitions because the incident light has zero linear momentum along the nanotube axis (i.e., the y direction). The second factor in Eq. (15) immediately leads to the selection rule

$$\kappa_{n'} - \kappa_n = \frac{2\pi l}{L}. \tag{16}$$

However, since $\kappa_{n'} - \kappa_n = (2\pi/L)(n' - n)$ for any ν, this is equivalent to

$$\Delta n = n' - n = l. \tag{17}$$

Physically, this condition ensures the conservation of angular momentum.

The matrix element $(s, n, k|\hat{\sigma}_x|s', n', k)$ appearing in Eq. (15) can be exactly evaluated at $k = 0$ because, as noted earlier, $|s, n, 0)$ is an eigenfunction of $\hat{\sigma}_x$ with an eigenvalue $\sigma_x = s\kappa_n/|\kappa_n| = \pm 1$:

$$\hat{\sigma}_x \frac{1}{\sqrt{2}} \begin{pmatrix} 1 \\ 1 \end{pmatrix} = \frac{1}{\sqrt{2}} \begin{pmatrix} 1 \\ 1 \end{pmatrix} \tag{18}$$

$$\hat{\sigma}_x \frac{1}{\sqrt{2}} \begin{pmatrix} -1 \\ 1 \end{pmatrix} = -\frac{1}{\sqrt{2}} \begin{pmatrix} -1 \\ 1 \end{pmatrix} \tag{19}$$

$$\frac{1}{\sqrt{2}} \begin{pmatrix} 1 & 1 \end{pmatrix} \cdot \frac{1}{\sqrt{2}} \begin{pmatrix} -1 \\ 1 \end{pmatrix} = 0. \tag{20}$$

Therefore, $(s, n, 0|\hat{\sigma}_x|s', n + l, 0)$ is non-zero only when the two states have the same value of σ_x, which is the second selection rule for optical transitions in SWCNTs. This rule is strictly true only when $k = 0$.

In summary, for an optical transition to occur in SWCNTs for light polarized perpendicular to the nanotube axis, the following two have to be satisfied:

1. $\Delta n = \pm 1$. Namely, the angular momentum quantum number has to change by ± 1. Here, $+1$ (-1) corresponds to right (left) circularly polarization.

2. $\Delta \sigma_x = 0$. Namely, the eigenvalue of the Pauli matrix $\hat{\sigma}_x$ has to be conserved. Strictly speaking, this rule applies only at $k = 0$.

Allowed transitions for a (17,0) nanotube

Here, we specifically consider a $\nu = -1$ nanotube, such as a (17,0) nanotube, which is semiconducting. Supplementary Table 1 summarizes the characteristics of the lowest four subbands in the conduction and valance bands. Here, $s = \pm 1$ is the band index, n is the angular momentum quantum number, κ_n is the quantized wavenumber along the circumference [see Eq. (4)], $\sigma_x = s\kappa_n/|\kappa_n|$, and $\varepsilon_{s,n}(0)$ is the band-edge ($k = 0$) energy.

Based on these values of quantum numbers and the selection rules we derived in Supplementary Note 1, we can conclude that the following transitions are possible for a $\nu = -1$ nanotube:

1. *Intersubband (or intraband)* transitions ($\Delta s = 0$): $C_{S1} \to C_{S3}$, $C_{S2} \to C_{S4}$, $V_{S1} \to V_{S3}$, and $V_{S2} \to V_{S4}$

2. *Interband* transitions ($\Delta s = 2$): $V_{S1} \to C_{S2}$ and $V_{S2} \to C_{S1}$

Note that all these six transitions universally occur at $(3/2)\varepsilon_0$, as mentioned in the main text.

	s	n	κ_n	σ_x	$\varepsilon_{s,n}(0)$		s	n	κ_n	σ_x	$\varepsilon_{s,n}(0)$
$\mathrm{C_{S1}}$	$+1$	0	$\frac{2\pi}{3L}$	$+1$	$\frac{\varepsilon_0}{2}$	$\mathrm{V_{S1}}$	-1	0	$\frac{2\pi}{3L}$	-1	$-\frac{\varepsilon_0}{2}$
$\mathrm{C_{S2}}$	$+1$	-1	$-\frac{4\pi}{3L}$	-1	ε_0	$\mathrm{V_{S2}}$	-1	-1	$-\frac{4\pi}{3L}$	$+1$	$-\varepsilon_0$
$\mathrm{C_{S3}}$	$+1$	$+1$	$\frac{8\pi}{3L}$	$+1$	$2\varepsilon_0$	$\mathrm{V_{S3}}$	-1	$+1$	$\frac{8\pi}{3L}$	-1	$-2\varepsilon_0$
$\mathrm{C_{S4}}$	$+1$	-2	$-\frac{10\pi}{3L}$	-1	$\frac{5}{2}\varepsilon_0$	$\mathrm{V_{S4}}$	-1	-2	$-\frac{10\pi}{3L}$	$+1$	$-\frac{5}{2}\varepsilon_0$

Supplementary Table 1: The characteristics of the lowest four subbands in the conduction and valance bands in a (17,0) carbon nanotube.

Allowed transitions for a (10,10) nanotube

Here, we specifically consider a $\nu = 0$ nanotube, such as a (10,10) nanotube, which is metallic. Supplementary Table 2 summarizes the characteristics of the lowest-lying subbands in the conduction and valance bands. Here, $s = \pm 1$ is the band index, n is the angular momentum quantum number, κ_n is the quantized wavenumber along the circumference [see Eq. (5)], $\sigma_x = s\kappa_n/|\kappa_n|$, and $\varepsilon_{s,n}(0)$ is the band-edge ($k = 0$) energy. It should be noted that the lowest subbands in metallic tubes, $\mathrm{C_{M0}}$ and $\mathrm{V_{M0}}$, are exceptional in the sense that their wavefunctions are eigenfunctions of $\hat{\sigma}_y$, rather than $\hat{\sigma}_x$.

Based on these values of quantum numbers and the selection rules we derived in Supplementary Note 1, we can conclude that the following transitions are possible for a $\nu = 0$ nanotube:

1. *Intersubband (or intraband)* transitions ($\Delta s = 0$): $\mathrm{C_{M1}^{n=+1}} \to \mathrm{C_{M2}^{n=+2}}$, $\mathrm{C_{M1}^{n=-1}} \to \mathrm{C_{M2}^{n=-2}}$, $\mathrm{V_{M1}^{n=+1}} \to \mathrm{V_{M2}^{n=+2}}$, and $\mathrm{V_{S2}^{n=-1}} \to \mathrm{V_{S4}^{n=-2}}$. Again, all these transitions universally occur at $(3/2)\varepsilon_0$.

2. *Interband* transitions ($\Delta s = 2$): $\mathrm{V_{M1}} \to \mathrm{C_{M2}}$ and $\mathrm{V_{M2}} \to \mathrm{C_{M1}}$. These transitions occur at $(9/2)\varepsilon_0$.

	s	n	κ_n	σ_x	$\varepsilon_{s,n}(0)$		s	n	κ_n	σ_x	$\varepsilon_{s,n}(0)$
$\mathrm{C_{M0}}$	$+1$	0	0	N/A	0	$\mathrm{V_{M0}}$	-1	0	0	N/A	0
$\mathrm{C_{M1}}$	$+1$	± 1	$\pm\frac{2\pi}{L}$	± 1	$\frac{3}{2}\varepsilon_0$	$\mathrm{V_{M1}}$	-1	± 1	$\pm\frac{2\pi}{L}$	∓ 1	$-\frac{3}{2}\varepsilon_0$
$\mathrm{C_{M2}}$	$+1$	± 2	$\pm\frac{4\pi}{L}$	± 1	$3\varepsilon_0$	$\mathrm{V_{M2}}$	-1	± 2	$\pm\frac{4\pi}{L}$	∓ 1	$-3\varepsilon_0$

Supplementary Table 2: The characteristics of the lowest four subbands in the conduction and valance bands in a (10,10) carbon nanotube.

Supplementary Note 2   Additional Experimental Data

Detailed gate-voltage dependence of the ISBP peak

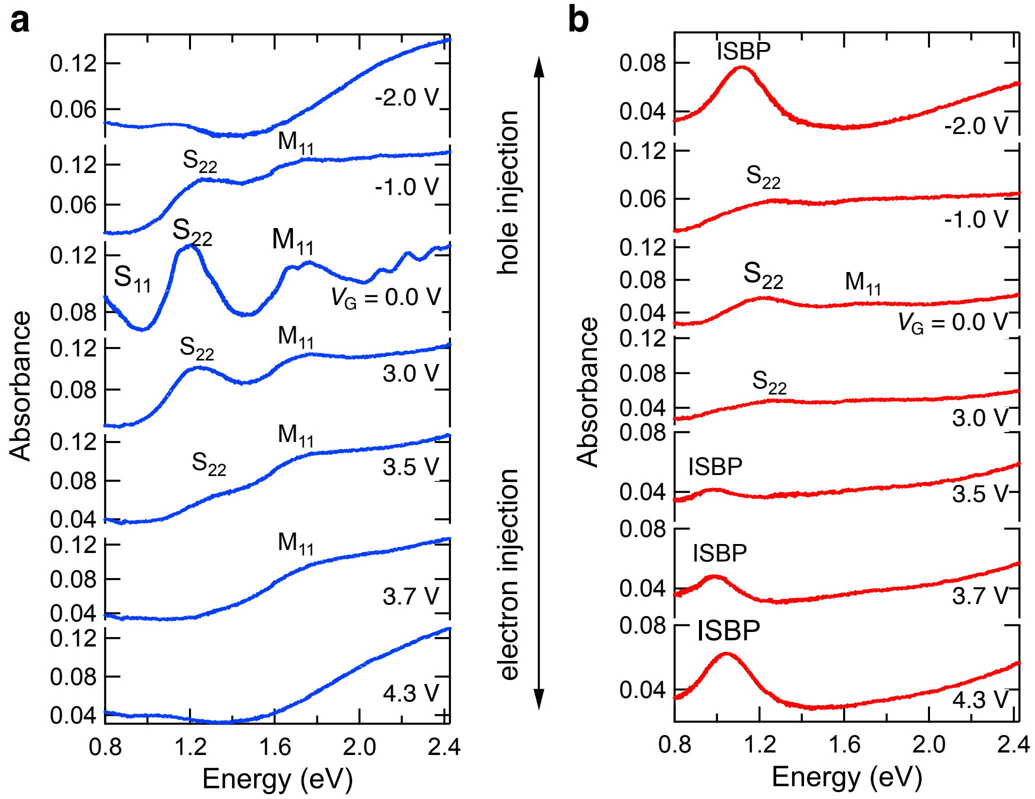

Supplementary Figure 1: **Appearance of the intersubband plasmon (ISBP) peak in a gated and aligned carbon nanotube film in polarized absorption spectra. a**, Parallel-polarization and **b**, perpendicular-polarization optical absorption spectra at various gate voltages for an aligned single-wall carbon nanotube film containing semiconducting and metallic nanotubes with an average diameter of 1.4 nm.

Gate-voltage-dependent linear dichroism spectra

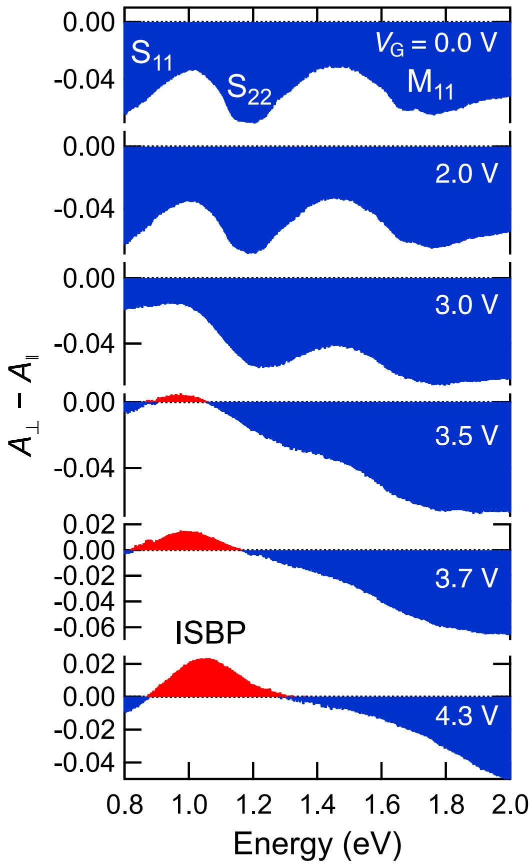

Supplementary Figure 2: **Linear dichroism spectra for a film of aligned unsorted SWCNTs with an average diameter of 1.4 nm.** Red and blue parts indicate positive (perpendicular polarization absorption being stronger) and negative (parallel polarization absorption being stronger) regions, respectively.

Spectra for metal- and semiconductor-enriched films

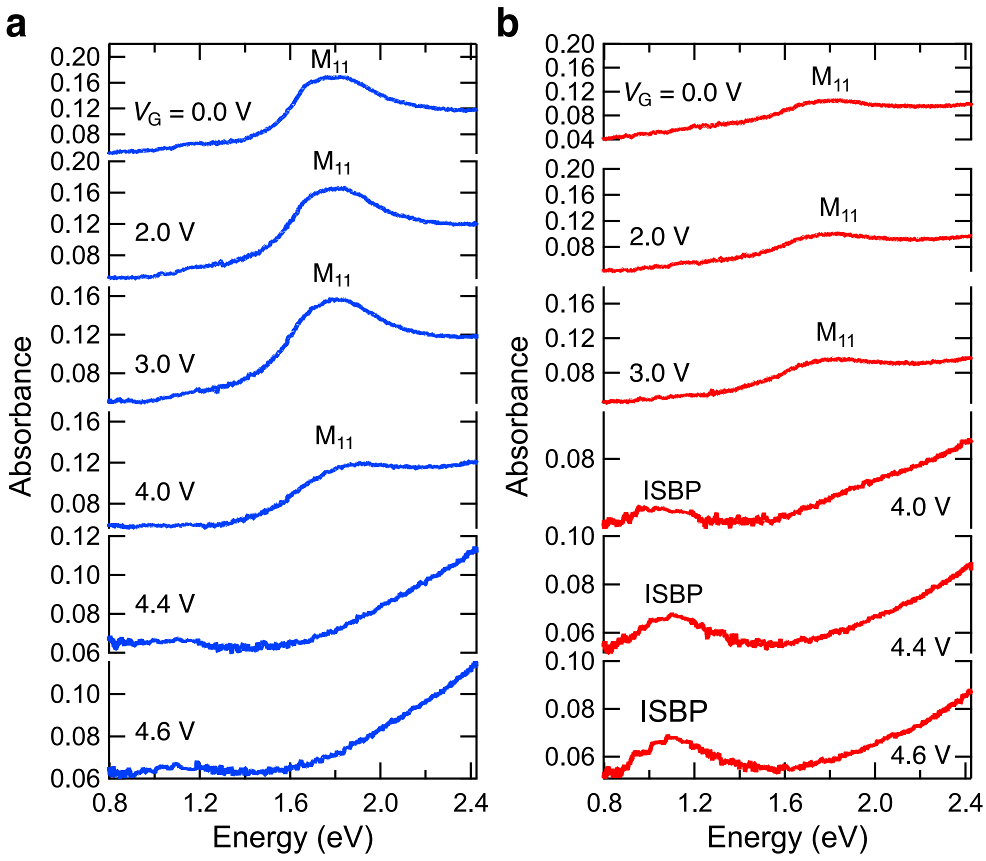

Supplementary Figure 3: **Polarization-dependent absorbance spectra for an aligned metallic SWCNT film at various gated voltages. a**, Parallel (blue lines) and **b**, perpendicular (red lines) polarization optical absorption for an aligned metallic SWCNT film with an average diameter of 1.4 nm at various values of gate voltage, V_G.

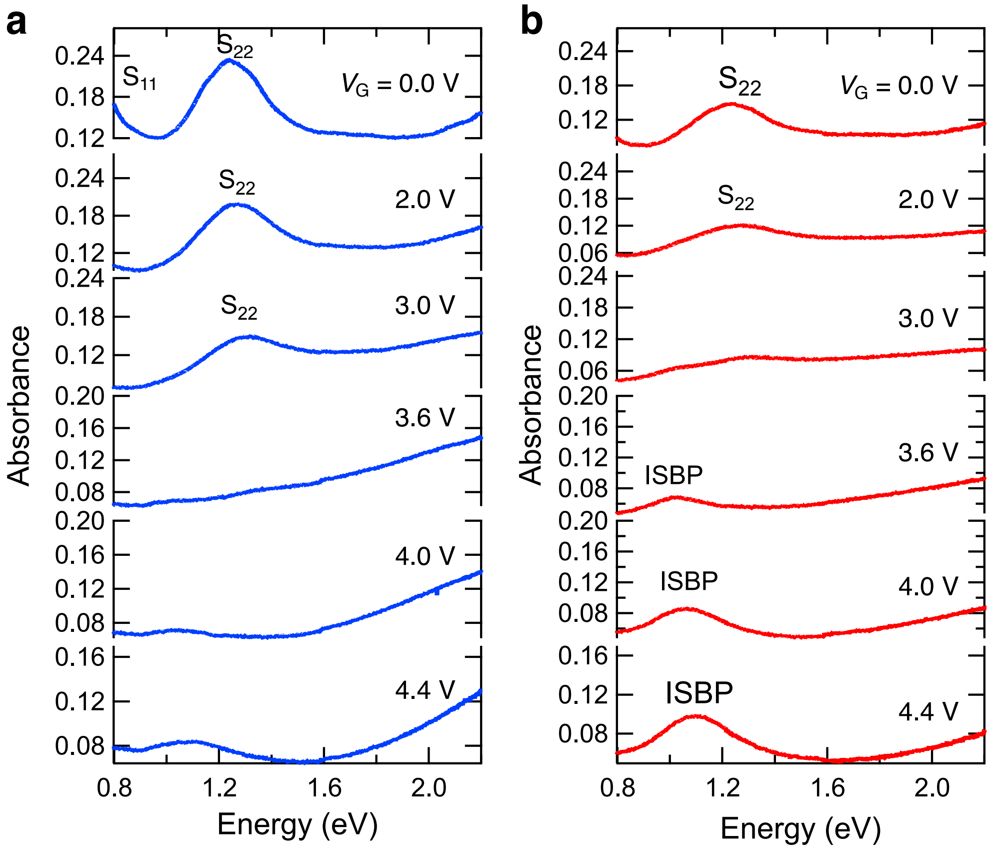

Supplementary Figure 4: **Polarization-dependent absorbance spectra for an aligned semiconducting SWCNT film at various gated voltages.** **a**, Parallel (blue lines) and **b**, perpendicular (red lines) polarization optical absorption for an aligned semiconducting SWCNT film with an average diameter of 1.4 nm at various values of gate voltage, V_G.

Supplementary Note 3   Calculations of Absorption Spectra

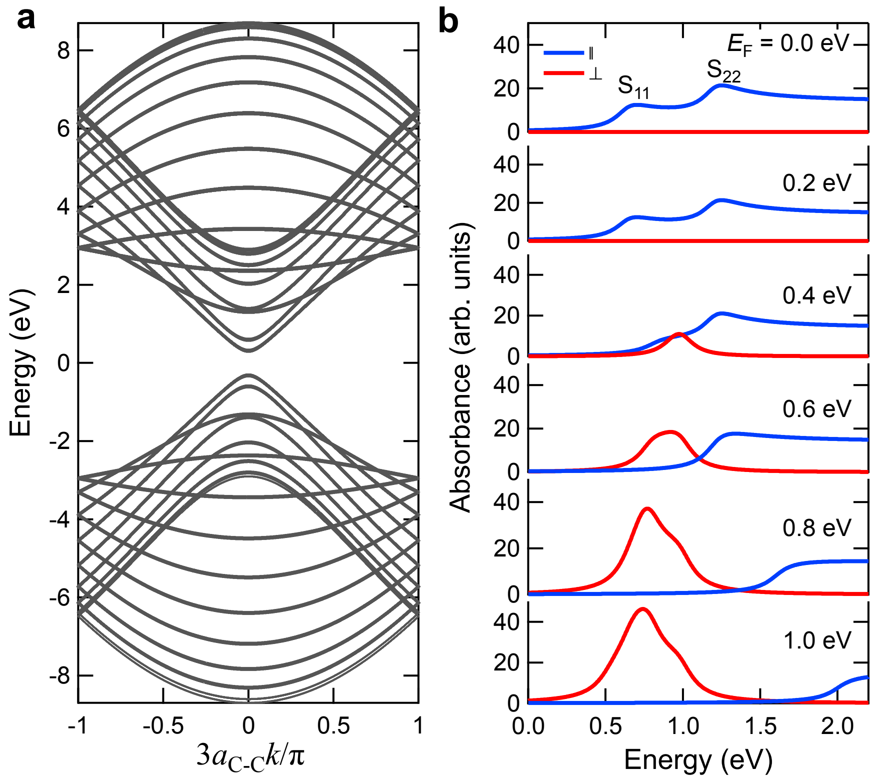

Supplementary Figure 5: **Calculated single-particle electronic band structure and absorbance spectra for a (17,0) SWCNT. a**, Band structure of a (17,0) SWCNT calculated using a tight-binding method with γ_0 = 2.9 eV. **b**, Calculated perpendicular (red line) and parallel polarization (blue line) optical absorption spectra for a (17,0) SWCNT at various values of Fermi energy E_F based on Equation (2) in the main text.

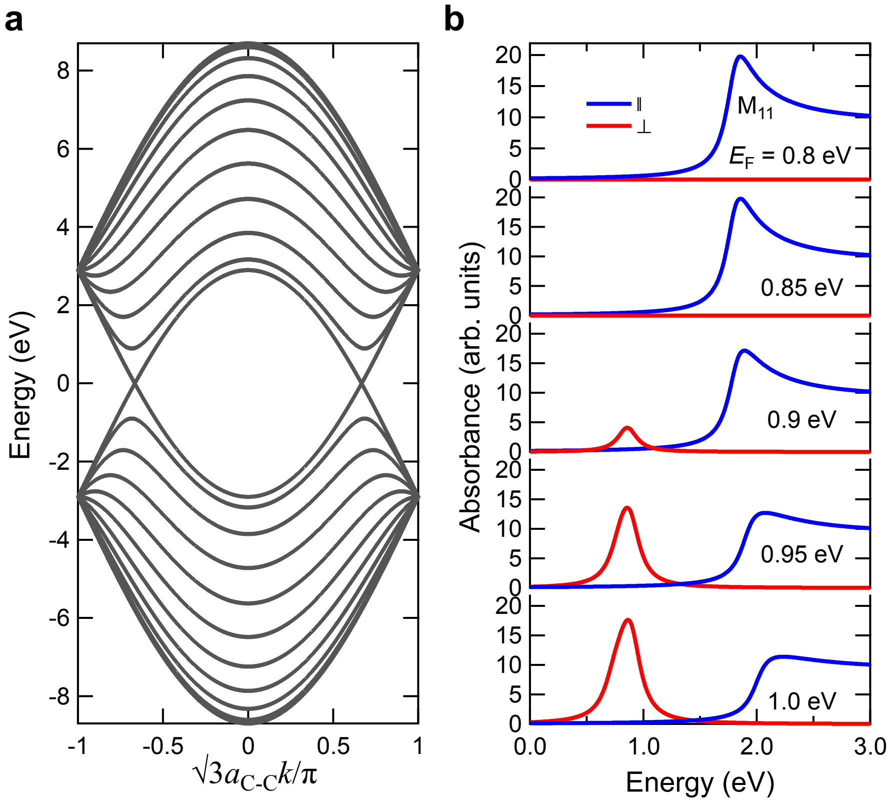

Supplementary Figure 6: **Calculated single-particle electronic band structure and absorbance spectra for a (10,10) SWCNT. a**, Band structure of a (10,10) SWCNT calculated using a tight-binding method with γ_0 = 2.9 eV. **b**, Calculated perpendicular (red line) and parallel polarization (blue line) optical absorption spectra for a (10,10) SWCNT at various values of Fermi energy E_F based on Equation (2) in the main text.

Supplementary Note 4 Drude Plasmon Model for SWCNTs

Based on the classical Drude model of plasmons, the perpendicular-polarization optical absorption in a doped SWCNT can be written as [2]

$$A_{\mathrm{p}}(\omega) = C\mathrm{Im}\left\{\frac{\omega_{\mathrm{g}}}{4\pi\omega_{\mathrm{g}}^2 - \omega(\omega + i\tau^{-1})}\right\}, \tag{21}$$

where C is a constant, $\omega_{\mathrm{g}} = (e/\hbar)(E_{\mathrm{F}}/\pi d_{\mathrm{t}})^{1/2}$, E_{F} is the Fermi energy, d_{t} is the nanotube diameter, and τ is a relaxation time. d_{t} was set to be 1.4 nm, and $\hbar/\tau$ was set to be 0.1 eV for our calculation. Note that the equations and quantities are expressed in atomic units, and we need to use $e^2/(\hbar c) \simeq 1/137$ to rewrite them in SI units. Thus, the resonance energy, $\hbar\omega_{\mathrm{p}}$, at which the peak of A_{p} occurs, is given by $2(\hbar c E_{\mathrm{F}}/137 d_{\mathrm{t}})^{1/2}$ as written in the main text.

Supplementary References

1. T. Ando, “Theory of Electronic States and Transport in Carbon Nanotubes,” J. Phys. Soc. Jpn. **74**, 777 (2005).

2. F. J. García de Abajo, “Graphene Plasmonics: Challenges and Opportunities,” ACS Photonics **1**, 135 (2014).
